# Editorial: Advances in cilia and flagella research

**DOI:** 10.3389/fcell.2025.1739712

**Published:** 2025-11-18

**Authors:** Maureen Wirschell, Lance Lee, Aimin Liu

**Affiliations:** 1 Division of Science and Math, McKendree University, Lebanon, IL, United States; 2 Sanford Research, Sioux Falls, SD, United States; 3 Department of Biology, Pennsylvania State University, University Park, PA, United States

**Keywords:** cilia, flagella, ciliopathies, gene expression, signaling

Cilia and flagella are small, microtubule-based organelles found on a wide range of eukaryotes, from single-celled protists to humans. These organelles protrude from the cell surface, acting as microscopic antennae that are critical for cellular signaling or generating movement to facilitate cell locomotion and fluid flow. There are two main classes; motile cilia and flagella that function in cell motility and have sensory functions, and primary (often immotile) cilia, which primarily perform sensory functions.

Cilia and flagella are highly conserved throughout evolution and are crucial structures for development and human health. Defects in ciliary assembly and/or function cause a broad array of pathologies collectively called “ciliopathies”, which can affect nearly every organ in the human body depending on the type of cilium and mutated gene.

Recent work has deepened our understanding of the molecular mechanisms that govern ciliogenesis and ciliary function in a wide range of model organisms. This Research Topic highlights recent advances in cilia and flagella biology, including topics covering ciliogenesis (Alanazi et al.; Brown; Stadler et al.), regulation of motility, regulation of cilia gene expression (Brown), and a review of a pivotal model organism that has pioneered cilia and flagella research, Chlamydomonas reinhardtii (Marshall).

In an exquisite review, Wallace Marshall details the benefits of using *C. reinhardtii* for studying cilia/flagella biology (Marshall). This unicellular green alga is a biflagellate protist that has played a leading role in the discovery of flagella composition, assembly, and regulation of function. *Chlamydomonas reinhardtii* is unparalleled in the range of experimental options offered for studying flagella, from classical genetics to biochemical analysis of isolated flagella, extensive genomic and proteomic resources, and recent advances in CRISPR gene editing.

Similarly, Jason Brown provides a review on historical and recent discoveries in the regulation of cilia gene expression (Brown). Ciliogenesis is accompanied by a rapid and highly coordinated transcriptional program involving hundreds of cilia genes. Early studies established *C. reinhardtii* and other simple eukaryotes like *Tetrahymena*, sea urchin, and Naegleria as viable model systems for studying cilia gene upregulation (Rosenbaum and Child, 1967; Vincensini et al., 2011; Fritz-Laylin and Fulton, 2016). Subsequent “omics” analyses have further revealed the molecular complexity of cilia and flagella gene networks, which consist of several hundreds of proteins with variations across cell types and organisms. Identification of target cilia genes and their kinetics in expression during ciliogenesis also remains a high priority. Identification of specific transcription factors that participate in these gene regulatory pathways include forkhead and RFX family members along with cis elements, such as the X-box, that further revealed the complexity in regulation of expression of cilia genes.

In addition to cilia gene transcription programs, cilia gene expression is also regulated by microRNAs primarily via post-transcriptional repression of target mRNAs (Alanazi et al.). The miR-34/449 family of miRNAs are highly expressed in ciliated cells and are important for regulating ciliogenesis and cilia length by targeting proteins like Cp110 (Song et al., 2014). Other miRNAs play roles in neurosensory organ development and cilia formation. Alanazi et al. revealed a role for miR-17 in ciliogenesis and cell cycle regulation and proliferation by examining the presence of miR-17 in primary cilia (Alanazi et al.). Their studies revealed a hyper-proliferative effect in cilia protein knock-out cells and suggested a regulatory role for miR-17 in promoting cell proliferation. These studies propose that primary cilia can serve as cellular compartments to sequester genetic material for regulation of gene expression. They also expand our understanding of ciliogenesis regulatory mechanisms from genetics to microRNAs.

Effective studies of cilia and flagella require reliable and reproducible tools and methods. In their manuscript, Haenseler et al. performed a systematic evaluation of ciliation rates and cilia length in neural stem cells, immature, and mature neurons differentiated from human induced pluripotent stem cells (hiPSCs) by various protocols (Haenseler et al.). Importantly, their studies determined that ciliation rate varied significantly between cell lines and differentiation methods, with ciliation rates decreasing with neuronal maturation and cell density. Moreover, cilia protein content also varied depending on maturation stage. These kinds of studies are pivotal for revealing differences in cilia at varying stages of development and across differentiation protocols.

Continued advances in high-resolution structural methods have revealed unprecedented detail in ciliary axoneme structure and ciliary protein structure (Bui et al., 2011; Pinskey et al., 2022; Gao et al., 2024; Schrad et al., 2025). Protein structure–function studies are paramount to understanding disease-causing mutations in ciliopathies (McCafferty et al., 2025). The Cilia and Flagella Associated Protein 410 (CFAP410) localizes to the basal body where it functions in ciliogenesis (Stadler et al., 2024; Stadler et al.). Mutations in the human CFAP410 gene result in skeletal and/or retinal ciliopathies (Wheway, et al., 2015; Suga et al., 2016; Wang et al., 2016; McInerney-Leo et al., 2017). Stadler et al. presents a 1.0-Å resolution crystal structure of the N-terminal domain of *T. brucei* CFAP410, highlighting the utility of *Trypanosoma brucei* as a model system for cilia structural studies. CFAP410 proteins have a bimodular design with two conserved domains. Disease-causing mutations in the N-terminal domain destabilize the CFAP410 structure, which likely disrupt its interactions with binding partners like NEK1 and provide a possible molecular model for how CFAP410 mutations cause ciliopathy phenotypes.

Considering that primary cilia were deemed vestigial organelles until the end of the 20th century, and little was known during that time about the mechanisms that influence motile cilia beating, the cilia field has made significant advancements in recent years. This Research Topic showcases the latest advances in the study of cilia and flagella, now known to be ubiquitous organelles that play vital roles in motility and sensory perception. The articles and reviews presented here deepen our understanding of their molecular composition, assembly, and functions in motility, development, and normal cell and tissue physiology. Continued research is needed to fully reveal the intricate interplay between cilia organelles, fundamental biological processes, and the impact of pathogenic mutations on ciliopathy etiology. Key discoveries in these areas will uncover new insights that could lead to novel therapies for diseases rooted in ciliary dysfunction.
